# Molecular docking of bioactive compounds derived from *Moringa oleifera* with p53 protein in the apoptosis pathway of oral squamous cell carcinoma

**DOI:** 10.5808/gi.21062

**Published:** 2021-12-31

**Authors:** Sonali Rath, Manaswini Jagadeb, Ruchi Bhuyan

**Affiliations:** 1 Department of Medical Research Health Sciences, IMS and SUM Hospital, Siksha ‘O’ Anusandhan (Deemed to be) University, Bhubaneswar 751003, India; 2 Department of Bioinformatics, Centre for Post Graduate Studies, Odisha University of Agriculture and Technology, Bhubaneswar 751003, India; 3 Department of Oral Pathology and Microbiology and Department of Medical Research Health Sciences, IMS and SUM Hospital, Siksha ‘O’ Anusandhan (Deemed to be) University, Bhubaneswar 751003, India

**Keywords:** apoptosis pathway, bioactive compound, molecular docking, *Moringa oleifera*, oral squamous cell carcinoma, p53

## Abstract

*Moringa oleifera* is nowadays raising as the most preferred medicinal plant, as every part of the moringa plant has potential bioactive compounds which can be used as herbal medicines. Some bioactive compounds of *M. oleifera* possess potential anti-cancer properties which interact with the apoptosis protein p53 in cancer cell lines of oral squamous cell carcinoma. This research work focuses on the interaction among the selected bioactive compounds derived from *M. oleifera* with targeted apoptosis protein p53 from the apoptosis pathway to check whether the bioactive compound will induce apoptosis after the mutation in p53. To check the toxicity and drug-likeness of the selected bioactive compound derived from *M. oleifera* based on Lipinski’s Rule of Five. Detailed analysis of the 3D structure of apoptosis protein p53. To analyze protein’s active site by CASTp 3.0 server. Molecular docking and binding affinity were analyzed between protein p53 with selected bioactive compounds in order to find the most potential inhibitor against the target. This study shows the docking between the potential bioactive compounds with targeted apoptosis protein p53. Quercetin was the most potential bioactive compound whereas kaempferol shows poor affinity towards the targeted p53 protein in the apoptosis pathway. Thus, the objective of this research can provide an insight prediction towards *M. oleifera* derived bioactive compounds and target apoptosis protein p53 in the structural analysis for compound isolation and *in-vivo* experiments on the cancer cell line.

## Introduction

Oral squamous cell carcinoma (OSCC) is the most widely recognized oral malignant growth found in India and is the sixth most common cancer worldwide [1]. Chemotherapy can be considered as the backbone of several cancer treatments including OSCC but there are possibilities of death occurs if chemotherapeutic resistance leads to therapeutic failure [2]. There are studies in which researchers conveyed those natural bioactive compounds which are rich in flavonoids and polyphenols can reduce the risk of OSCC as they contain abundant phytochemicals which possess anti-cancer properties [3]. Thus, plants containing medicinal properties can be used for the treatment and management of OSCC.

In India, *Moringa oleifera* is rapidly gaining popularity as herbal medicine, due to its potential bioactive compounds which have anti-cancer properties. *M. oleifera* is popularly known as the “The Drumstick Tree” in India [4]. There are some studies where it is reported that bioactive compounds from *M. oleifera* interact with OSCC cell lines and inhibited cell proliferation which is similar to some anti-cancer drugs [5]. Thus, the bioactive compounds from *Moringa oleifera* could potentially induce programmed cell death by activating P53 tumor suppressor proteins and other associated proteins in the apoptosis pathway.

Several tumor suppressor genes, proto-oncogenes, and oncogenes are involved in OSCC. Mutation in p53 gene is one of the frequent phenomena in OSCC along with other cancers in human. Although, its function in tumorigenesis and its interrelation concerning prognosis is still under evaluation and indeterminate [6]. Hence OSCC cells show a lack of molecular targets, which is difficult for chemotherapeutical drugs to be effective. Those problems have led various scientists to do molecular docking studies to speed up anti-cancer research. As p53 is also known as tumor suppressor protein, its main function is to stop the OSCC progression by arresting the cell cycle through p21, DNA repair regulation with base excision repair activity, and inducing apoptosis by activation of both extrinsic and intrinsic pathways [7].

**Fig. 1.**
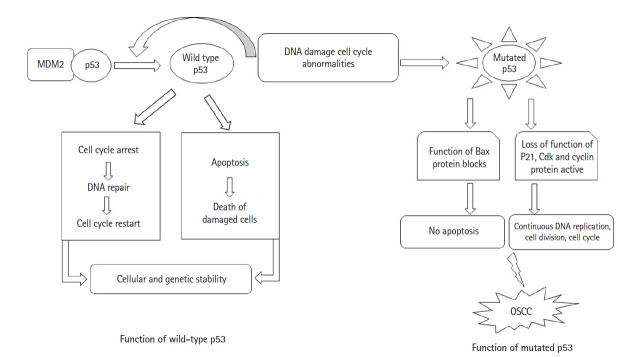
Function of wild-type p53 and mutated p53. OSCC, oral squamous cell carcinoma.

In most of the OSCC cases, 65%‒85% mutation in p53 protein is reported [8]. Loss of function as tumor suppressor protein occurs after mutation in p53, and gain function as a proto-oncogene, thus clinically increase cancer cell progression and tumorigenesis [9-11]. Dominant-negative activity through oligomerization, mutated p53 can inactivate the normally functioning wild-type p53 [7]. In OSCC, overexpression of mutated p53 found resistant to several chemotherapy drugs which are used in treatment such as cisplatin, doxorubicin, temozolomide, tamoxifen, cetuximab, and gemcitabine [12]. Some studies also showed that there are five hotspot codons in p53 protein which decrease the sensitivity of Cisplatin-based on chemotherapy, which results in worse outcomes clinically [13,14]. Hence for the prognosis and appropriate treatment for OSCC in the future the mutational status of p53 should be known (Fig. 1).

The docking between protein and ligand, ligand-binding mechanism, and the perception about the most stable complex of protein-ligand can be identified by using molecular docking studies [15]. In *in-silico* analysis, targeting the apoptosis pathway to overcome the drug resistance common to OSCC chemotherapy can be a promising approach towards drug development and discovery. OSCC cells with p53 alteration enter into the synthesis phase and synthesize the damaged DNA. Hence these cells fail to enters into the apoptosis pathway which results in the progressive appearance of cells with damaged DNA and clone of OSCC cells that evolve into more aggressive carcinoma [16]. So, targeting p53 with a drug made from a bioactive compound can trigger apoptosis.

This study determines the interaction between the bioactive compounds derived from *M. oleifera* and tumor suppressor protein p53 in the apoptosis pathway. Based on prior studies the bioactive compounds from *M. oleifera* were selected and validated with toxicity, drug-likeness, and Lipinski’s Rule of Five. The compounds were further examined by molecular docking studies in order to evaluate their binding affinity towards p53 protein in the apoptosis pathway.

## Methods

### Protein model, tertiary structure and prediction of active site

**Fig. 2.**
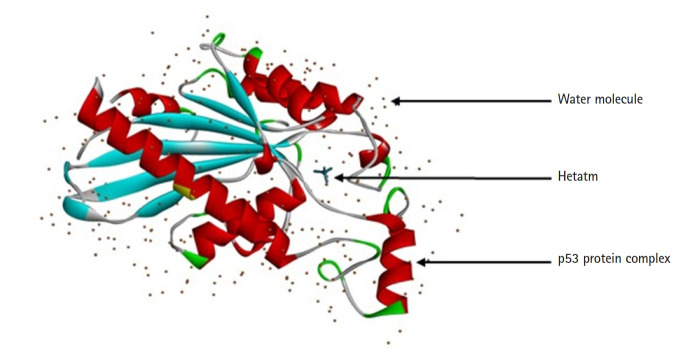
3D structure of p53-induced glycolysis and apoptosis regulator protein from *Homo sapiens*.

The protein structure of P53 (PDB ID: 3DCY) was fetched from the Research Collaboratory for Structural Bioinformatics‒Protein Data Bank (RCSB-PDB) [17]. Protein data was saved in .pdb file format. after removal of side chains, water, hetatm, and ligand molecules using BIOVIA Discovery Studio 2021 Client [18]. The tertiary structure of a protein is predicted with PyMOL 2.5.0. CASTp server predicts the active sites present in the structure of protein, which was used to evaluate the protein [19] (Fig. 2).

### Selection of compounds

**Fig. 3.**
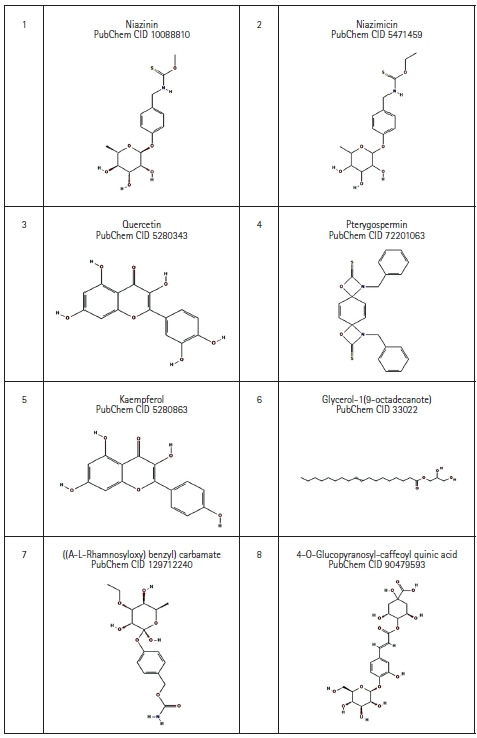
2D structure of *Moringa oleifera* derived bioactive compound retrieved from PubChem database.

Eight bioactive compounds from *M. oleifera* were selected which possess anti-cancer property based on literature review [4,20,21]. The 3D structures of those compounds were downloaded with help of PubChem database in SDF file format, which then was converted to .pdb file format using the BIOVIA Discovery Studio 2021 Client [18] (Fig. 3).

### Toxicity and drug likeness prediction

The drug-likeness is the consistency of orally active drugs which can be determined by Lipinski’s Rule of Five [22,23]. It anticipates the infusion or incorporation of a compound when the value of calculated logP (ClogP) > 5.37, molecular weight (MW) > 500 g/mol, more than 10 acceptors and more than five donors of H- bond [24].

The selection of compounds can be done by determining the drug score. The bioactive compounds having greater drug score values are referred to as better drug candidates [25]. Swiss ADME predictor was used for the screening of the bioactive compounds in this study. It gives the details of the bioactive compounds like the number of rotatable bonds, hydrogen acceptors, and hydrogen donors. The analyzed compounds were screened with Lipinski’s Rule of Five and the molecular docking study can be done with the compounds without any violation [26].

### Molecular docking

Molecular docking analysis was done with the tool known as Auto Dock Tools 1.5.6 [27]. To the structure of protein Polar hydrogen atoms and Gasteiger partial charges were incorporated. The structure of the protein was then saved in .pdbqt file format for analysis. Active sites of protein which are predicted by using the CASTp server were selected and accordingly the grid box was set.

For docking study, Lamarckian Genetic Algorithm 4.2 was used [28] and the protein macromolecule was kept rigid throughout the docking study. Genetic algorithm runs were set at 30 and the other parameters were left as default settings for docking analysis. The best protein-ligand conformation was chosen from Auto Dock 1.5.6 scoring function, and they have ranked accordingly to their binding affinities. BIOVIA Discovery Studio 2021 Client, PyMOL 2.5.0 was used for post docking analysis [29].

## Results

### Tertiary structure analysis

**Fig. 4.**
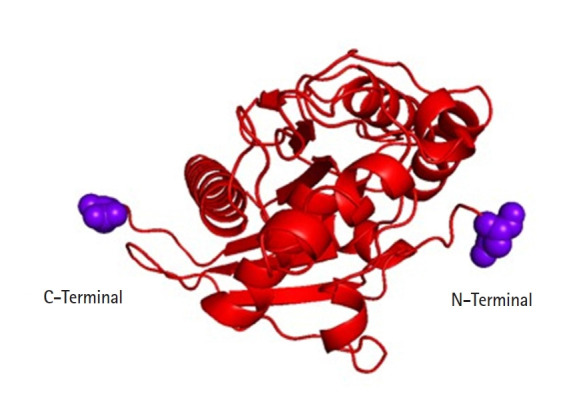
Tertiary structure of targeted p53 protein.

On chromosome 17p 13.1 of humans, the p53 gene is located which encodes a 53-kDa, 393 AA, and nuclear phosphoproteins known for cell proliferation and regulation of cell growth [30]. Three hundred ninety-three amino acids of the human p53 protein contains four major functional domains. At the C-terminal portion there is an oligomerization domain (AAs 323–356) and a regulatory domain (AAs 360–393). And the N-terminus is a transcriptional activation domain (AAs 1–42) and within the central part of p53 is the sequence-specific DNA-binding domain (AAs 102–292). The amino acid asparagine (ASN) is present in both ends of the targeted p53 protein [31] (Fig. 4).

### Prediction of drug-likeness

Swiss ADME web server predicts the physiochemical factors of the ligands as shown in Table 1. Lipinski’s Rule of Five was used for filtration and screening of ligands, and eight potential active bioactive compounds with anti-cancer properties were left as it is. Generally, Lipinski’s Rule states that an orally active drug cannot violate more than one of Lipinski’s Rule parameters. One among the eight bioactive compounds that don’t satisfy Lipinski’s Rule, the one that did not satisfy Lipinski’s Rule is 4-O-glucopyranosyl-caffeoyl quinic acid/4-O-(4'-o-alpha-D-glucopyranosyl)-caffeoyl quinic acid with three violations as MW is >500 g/mol, i.e., 516.45 g/mol; the number of H- donor is >5, i.e., 9 and number of H-acceptor is >10, i.e. 14. All other bioactive compounds satisfy Lipinski’s Rule of Five. After screening with Lipinski’s Rule of Five, seven compounds were taken for further the docking study (Table 1).

### Active-site prediction

**Fig. 5.**
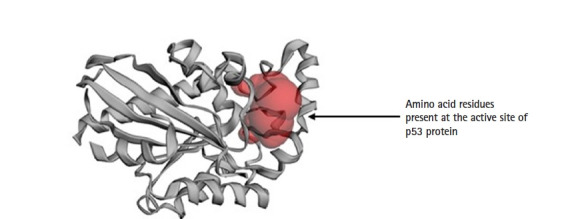
Apoptosis protein p53 with active amino acid site.

**Fig. 6.**
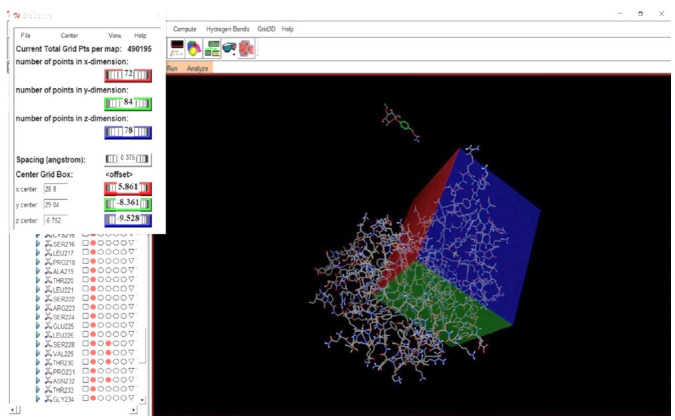
Grid-box placing at the active site of p53 protein.

The active site prediction of targeted p53 protein was done with CASTp 3.0. At the predicted active site of protein p53 there are 28 AA residues. The grid box was centered at the predicted active site for the further docking study (Table 2, Figs. 5 and 6).

### Molecular docking

The targeted p53 protein is docked with seven potential bioactive compounds which are validated by Swiss ADME. Table 3 indicates the binding affinity for each compound with targeted apoptosis protein. The docking pattern of each bioactive compound after docking with p53 was analyzed using AutoDock 1.5.6 and the docking of the bioactive compounds with active site residues of protein was analyzed using PyMol 2.5.0 and Discovery studio Visualizer 4.1 client. The bioactive compounds showed varying degrees of favorable docking with the targeted protein p53. As 4-O-glucopyranosyl-caffeoyl quinic acid/4-O-(4'-o-alpha-D-Glucopyranosyl)-caffeoyl quinic acid had three violations and does not satisfy Lipinski’s Rule, no further docking study was done with this compound. After docking it is found that quercetin had a high binding affinity towards p53 is ‒6.72 whereas the lowest binding affinity detected was -5.29 for kaempferol towards p53 (Table 3).

**Fig. 7.**
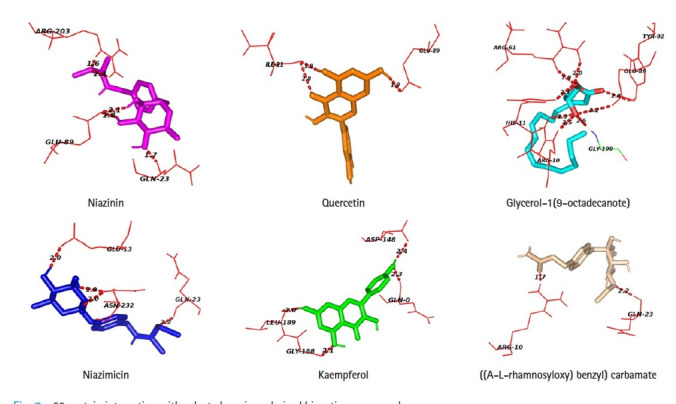
p53 protein interacting with selected moringa derived bioactive compounds.

Protein-ligand docking analysis showed that quercetin had an adequate binding affinity towards p53. There are 3 H-bonding was observed between p53 and quercetin; two bonds with ILE- 21 and one bond with GLU- 89. Niazinin, niazimicin, glycerol-1(9-octadecanote) and ((A-L-rhamnosyloxy) benzyl) carbamate forms five, four, eight, and two hydrogen bonds respectively. Kaempferol shows the lowest binging affinity towards p53, hence formed four H- bonds with GLN-0, ASP-148, GLY-188, and LEU-189 residues of the active site of p53 (Table 4, Fig. 7).

## Discussion

As per Lipinski’s Rule of Five, the weight of a compound is needed to be <500 g/mol, lipophilicity (iLogP) < 5, H-bond donors should be 5 or <5 and H-bond acceptors must be 10 or < 10. These parameters are remarkably associated with intestinal permeability and dissolvable in the first step of oral bioavailability. The molecule can’t be a drug molecule if the number of violence of law is more than two [23]. If a bioactive compound fails according to the parameters of Lipinski’s Rule of Five, it will cause difficulty if consumed. The molecular characteristics of a drug’s pharmacokinetics of the body can be explained by the parameters of ADME.

The compounds which are selected for the docking analysis have lipophilicity (iLogP) less than five while niazinin, niazimicin, quercetin, kaempferol, glycerol-1(9-octadecanote), ((A-L-rhamnosyloxy) benzyl) carbamate had less than 5 H-bond donors (Table 1). 4-O-(4'-o-Alpha-D-glucopyranosyl)-caffeoyl quinic acid has MW > 500 g/mol, >5 H-bond donors and >10 H-bond acceptors. Whereas Pterygospermin has no H-bonding with p53 protein. These data represent the violation of Lipinski’s Rule and docking analysis was done with the compounds which obey Lipinski’s Rule. Toxicity is an important constituent that often surpasses the ADME parameters. Due to unfavorable effects created from the toxicity leads to the breakdown of the drugs at the clinical trial [32].

The results of molecular docking generate the binding energy between the protein and ligand which is an essential parameter. This provides information about the binding affinity and strength of protein and ligand-receptor docking. The lower the binding energy value, the higher is the binding affinity and docking. This research indicates the binding energies of the selected apoptosis protein p53 with *M. oleifera* derived bioactive compounds and amino acid residues were identified, which take part in the binding dockings through molecular docking analysis.

The bioactive compound which exhibits the best binding affinity with p53 is quercetin with ‒6.72 kcal/mol. Prior studies show that the bioactive compounds derived from various parts of *M. oleifera* lower the proliferation of malignant cells, which also causes the death of cancerous cells, approximately 20%‒22%. It causes apoptosis at the growth phase 1 (G1) and induced cell arrest at growth phase 2 (G2) or mitosis phase (M). The bioactive compounds increased the p53 protein level in the cell [4].

Glycerol-1(9-octadecanote) forms the highest number of hydrogen bonds during protein-ligand docking. Niazinin possesses five interacting bonds with the amino acids GLN-23, GLU-89, ARG-203 at a bond distance of 1.7, 1.8, 2.1, 1.6, and 2.4 Å. In the case of glycerol-1(9-octadecanote), ARG-10 forms double docking at the distance of 2.3 and 2.5 Å. Niazimicin and kaempferol have a similar number of hydrogen bonds but niazimicin bonds are closer than kaempferol.

In summary, this study indicated that quercetin shows the best docking with targeted p53 while other compounds show comparatively lower affinities towards the targeted protein. However, if we use different software or tool for the analysis the outcome may differ as different applications use different algorithms [33]. Adding to this, the protein data which was submitted to different databases earlier may differ from each other as the methodologies carried out may be different [34]. Future expects for this study includes the comparison of the retrieved protein structure from different databases with the different experimental models, tools, and software. Furthermore, the *in-vitro* and *in-vivo* studies of protein and gene expression must be evaluated in order to support the *in-silico* results.

There are so many risk factors for OSCC to happen but the main cause of it is the damaged area losses its ability to repair. As a result, tumor formation cannot be prevented. Sometimes the targeted protein p53 which is a tumor suppressor protein also called apoptosis protein when get mutated fails to enter into the apoptosis pathway, as a result, the damaged DNA gets synthesized and it results in OSCC. Nowadays herbal medication is more preferable so we took the *Moringa oleifera* derived bioactive compounds which possess anti-cancer properties. In this research, we concluded that quercetin had a good docking with the targeted p53 protein whereas kaempferol showed poor affinity towards the protein. There is no H-bonding between ptergyospermin and the targeted protein p53. The other bioactive compounds as niazinin, niazimicin, glycerol-1(9-octadecanote, ((A-L-rhamnosyloxy) benzyl) carbamate shows moderate affinity towards p53. This result provides valuable information about the bioactive compounds derived from *Moringa oleifera* that can be used for OSCC treatment to check the synthesis of targeted protein and to let the cell with damaged DNA undergo apoptosis.

## Figures and Tables

**Table 1.** Lipinski’s Rule for *M. oleifera* derived bioactive compounds by Swiss ADME server

Ligands	Lipinski’s Rule of Five					
	iLogP <5	Molecular wight (g/mol) <500	Hydrogen acceptor <10	Hydrogen donor <5	Drug-likeness Lipinski’s rule follows	Violation
Niazinin	2.55	343.40	6	4	Yes	0
Niazimicin	3.06	357.42	6	4	Yes	0
Quercetin	1.63	302.24	7	5	Yes	0
Ptergyospermin	3.45	406.52	2	0	Yes	0
Kaempferol	1.70	286.24	6	4	Yes	0
Glycerol-1(9-octadecanote)	4.33	356.54	4	2	Yes	0
((A-L-Rhamnosyloxy) benzyl) carbamate/O-Ethyl-4-(alpha-l-rhamnosyloxy) benzyl carbamate	2.63	357.36	8	4	Yes	0
4-O-Glucopyranosyl-caffeoyl quinic acid/4-O-(4'-o-alpha-D-Glucopyranosyl)-caffeoyl quinic acid	1.43	516.45	14	9	No	3

**Table 2.** Active site prediction of targeted protein p53 with CASTp 3.0 server

Protein	Volume (SA)	Area (SA)	Resolution	Total AA residue in chain A	AA residues at predicted active site
p53	410.952	380.470	1.75	275	28

SA, surface area; AA, amino acid.

**Table 3.** Dockings based on energy for selected bioactive compounds and targeted p53 protein

Compound	Binding affinity (kcal/mol)						
	Niazinin	Niazimicin	Quercetin	Ptergyospermin	Kaempferol	Glycerol-1(9-octadecanote)	((A-L-Rhamnosyloxy) benzyl) carbamate/O-Ethyl-4-(alpha-l-rhamnosyloxy) benzyl carbamate
Receptor p53	‒5.96	‒6.04	‒6.72	‒9.06	‒5.29	‒5.81	‒6.27

**Table 4.** Interacting amino acid residues of targeted p53 protein with selected bioactive compounds derived from *Moringa oleifera*

Targeted protein	Bioactive compound	No. of H-bond	Interacting residues	Distance (Å)
p53	Niazinin	5	GLN-23	1.7
			GLU-89	1.8
				2.1
			ARG-203	1.6
				2.4
	Niazimicin	4	GLU-13	2.0
			GLN-23	2.2
			ASN-232	1.9
				2.0
	Quercetin	3	ILE-21	1.9
				2.8
			GLU-89	1.9
	Ptergyospermin	0	-	-
	Kaempferol	4	GLN-0	2.3
			ASP-148	2.4
			GLY-188	2.1
			LEU-189	2.0
	Glycerol-1(9-octadecanote)	8	TYR-92	2.8
			GLU-89	3.2
			GLY-199	2.5
			ARG-10	2.3
				2.5
			HIS-11	2.1
			ARG-61	1.9
				2.0
	(A-L-Rhamnosyloxy) benzyl) carbamate/O-Ethyl-4-(alpha-l-rhamnosyloxy) benzyl carbamate	2	ARG-10	1.7
			GLN-23	2.2
